# Supplementary material for: Dependence of the Structural and Magnetic Properties on the Growth Sequence in Heterostructures Designed by YbFeO3 and BaFe12O19
Source: Nanomaterials (Basel). 2024 Apr 18;14(8):711. doi: 10.3390/nano14080711 (PMC11054277; doi:10.3390/nano14080711)
Supplement: Supplementary file 1 [file nanomaterials-14-00711-s001.zip › nanomaterials-2931345-supplementary.pdf]

nanomaterials

Supplementary Materials

Dependence of the Structural and Magnetic Properties on the Growth Sequence in Heterostructures Designed by $YbFeO_3$ and $BaFe_{12}O_{19}$

Sondes Bauer [1,*], Berkin Nergis [1], Xiaowei Jin [2], Reinhard Schneider [2], Di Wang [3,4], Christian Kübel [3,4], Petr Machovec [5], Lukas Horak [5], Vaclav Holy [5], Klaus Seemann [6], Tilo Baumbach [1,7] and Sven Ulrich [6]

[1] Institute for Photon Science and Synchrotron Radiation, Karlsruhe Institute of Technology, Hermann-von-Helmholtz-Platz 1, D-76344 Eggenstein-Leopoldshafen, Germany; berkin.nergis@kit.edu (B.N.); tilo.baumbach@kit.edu (T.B.)
[2] Laboratory for Electron Microscopy, Karlsruhe Institute of Technology, Engesserstr. 7, D-76131 Karlsruhe, Germany; carriorjin@gmail.com (X.J.); reinhard.schneider@kit.edu (R.S.)
[3] Institute of Nanotechnology, Karlsruhe Institute of Technology (KIT), Hermann-von-Helmholtz-Platz 1, D-76344 Eggenstein-Leopoldshafen, Germany; di.wang@kit.edu (D.W.); christian.kuebel@kit.edu (C.K.)
[4] Karlsruhe Nano Micro Facility , Karlsruhe Institute of Technology (KIT), Hermann-von-Helmholtz-Platz 1, D-76344 Eggenstein-Leopoldshafen, Germany
[5] Department of Condensed Matter Physics, Charles University, Ke Karlovu 5, 121 16 Prague, Czech Republic; petr.machovec@matfyz.cuni.cz (P.M.); lukas.horak@matfyz.cuni.cz (L.H.); vaclav.holy@matfyz.cuni.cz (V.H.)
[6] Institute for Applied Materials, Karlsruhe Institute of Technology, Hermann-von-Helmholtz-Platz 1, D-76344 Eggenstein-Leopoldshafen, Germany; klaus.seemann@kit.edu (K.S.); sven.ulrich@kit.edu (S.U.)
[7] Laboratory for Applications of Synchrotron Radiation, Karlsruhe Institute of Technology, Kaiserstr. 12, D-76131 Karlsruhe, Germany
* Correspondence: sondes.bauer@kit.edu

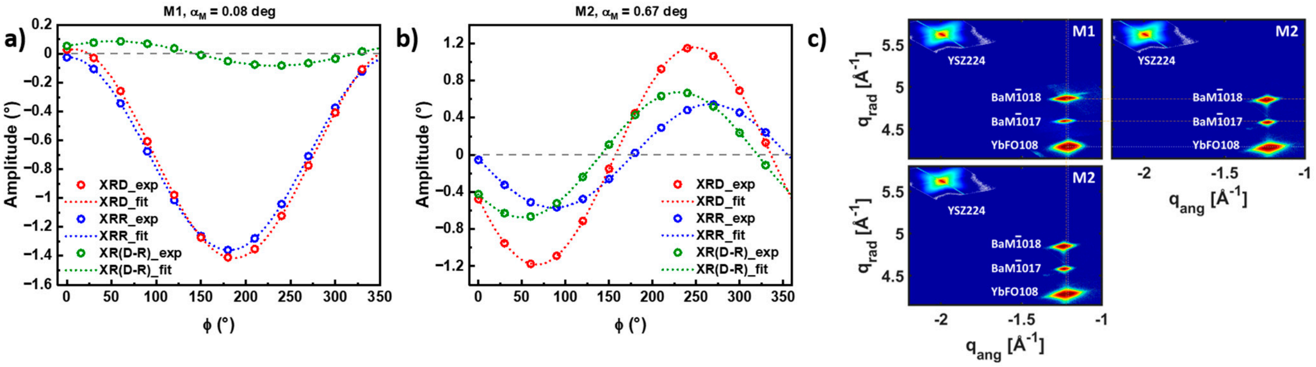

Figure S1. Inclination of the surface and YSZ crystal lattice in samples **a)** M1, **b)** M2. The blue points are the inclination of the surface, the red points the inclination of the crystal lattice of the YSZ substrate, and the green points the difference of the inclinations. e.g., the miscut in the given azimuths. The lines are fits of the experimental data. **c)** Reciprocal space maps of YSZ224, YSZ133, BaM-1017, BaM-1018 and YbFO108 of M1 and M2.

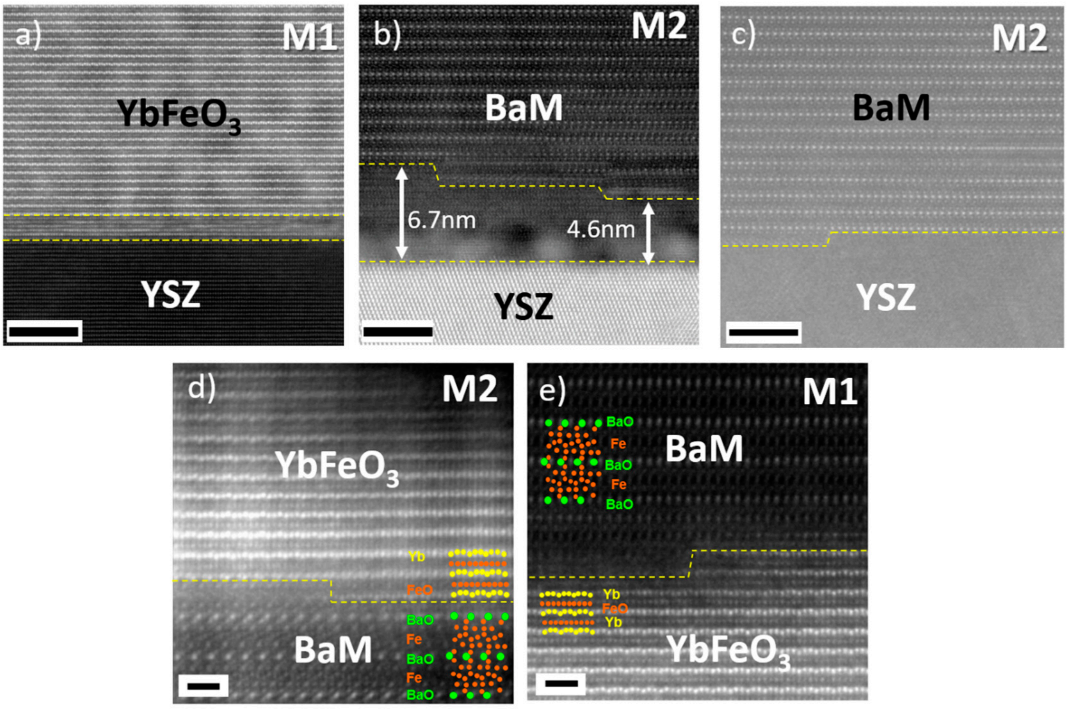

Figure S2. Additional atomic resolution HAADF images of the M1 and M2 systems focusing on the interfaces: **a)** YbFO/YSZ interface in the M1 system, highlighting an interlayer with a yellow dashed line, **b)** BaM/YSZ interface in the M2 system, where a significant interlayer varying from 4.6 to 6.7 nm is marked by yellow dashed lines along with atomic steps at the BaM interface, **c)** BaM/YSZ interface in M2 without an interlayer, with miscut-related YSZ surface steps indicated by a yellow dashed line, **d)** YbFO/BaM interface in M2, where interface atomic steps are outlined by a yellow dashed line, and individual atoms are identified by colored filled circles, and **e)** BaM/YSZ interface in M1, showcasing surface steps on YbFO with a yellow dashed line and atom identification using colored circles. The scale bars for (a–c) are 5nm and (d–e) are 1 nm in size.

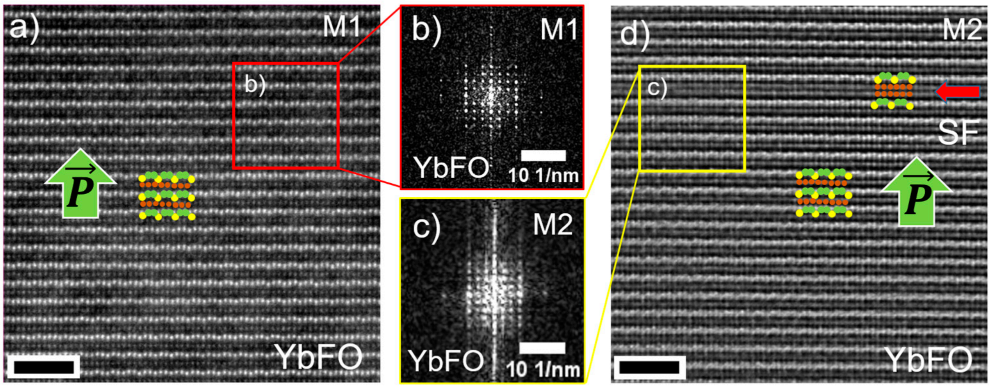

Figure S3. High resolution STEM HAADF images and diffractograms of YbFO layers in M1 and M2 systems. **(a,d)** HAADF images of YbFO layers in M1 and M2, respectively, illustrating the atomic arrangements with colour-coded spheres for Yb (green and yellow) and Fe (brown) atoms. Note a stacking defect in M2 (d). FFT analysis diffractograms of M1 **b)** and M2 **c)** confirming the ferroelectric metaphase of YbFeO3 in both layers. The scale bars for (a,d) are 2 nm in size.

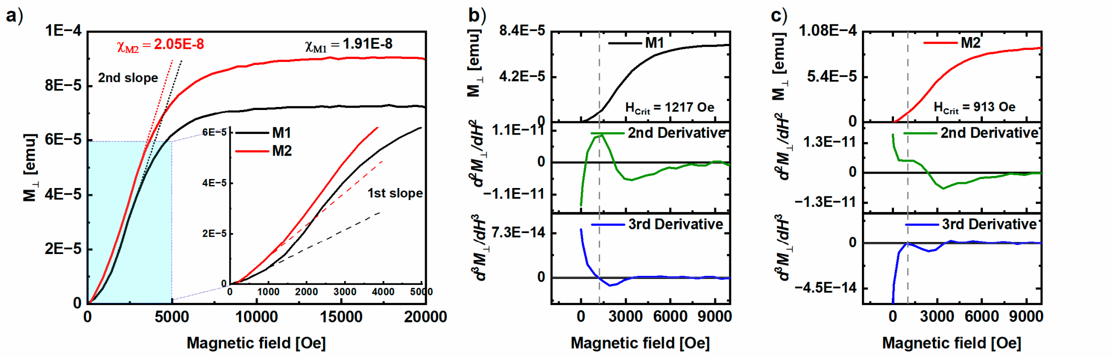

Figure S4. Initial magnetization and H_{crit} analysis for M1 and M2 heterostructures **a)** initial magnetization curves (IMC) for M1 and M2, with an inset detailing two-slope behavior and determination of H_{crit} (highlighted by dashed lines). **(b,c)** Initial magnetization and its derivatives for M1 and M2 respectively, H_{crit} at the zero point of the third derivative, H_{crit} is marked by vertical dashed line.
